# A Case Report of a Patient With Recalcitrant Pemphigus Vulgaris Treated With Rituximab

**DOI:** 10.1002/ccr3.73279

**Published:** 2026-07-31

**Authors:** Mahlatse Cordelia Kgokolo, Lesedi Makgwethele Nevondo, Michaela Beetge, Razia A. G. Khammissa

**Affiliations:** ^1^ Department of Dermatology, School of Medicine, Faculty of Health Sciences University of Pretoria Pretoria South Africa; ^2^ Department of Anatomical Pathology, School of Medicine, Faculty of Health Sciences University of Pretoria Pretoria South Africa; ^3^ Department of Periodontics and Oral Medicine, School of Dentistry, Faculty of Health Sciences University of Pretoria Pretoria South Africa

**Keywords:** autoimmune blistering diseases, corticosteroids, corticosteroid‐sparing drugs, pemphigus vulgaris, rituximab

## Abstract

Pemphigus vulgaris may demonstrate differential treatment responses across disease sites. In this case, cutaneous lesions responded to high‐dose corticosteroids, whereas refractory oral disease required rituximab to achieve remission, highlighting the need for individualized, multimodal therapy to achieve sustained disease control.

## 1 Introduction

Pemphigus vulgaris (PV) is a rare, potentially life‐threatening autoimmune blistering disease caused by IgG autoantibodies targeting desmoglein 3 (mucosal disease) or both desmoglein 1 and 3 (mucocutaneous disease), resulting in acantholysis, flaccid bullae, and erosions of the skin and mucous membranes [1, 2]. Oral mucosal involvement is the initial manifestation in the majority of patients, affecting approximately 90%, with the buccal and labial mucosa most commonly involved [3, 4]. Oral lesions are frequently refractory and carry significant morbidity, impairing nutrition, speech, and quality of life [2].

The disease most commonly presents between the fourth and sixth decades and is diagnosed histopathologically by suprabasal acantholysis, with direct immunofluorescence demonstrating IgG and C3 deposition on keratinocyte surfaces, and serologically by anti‐Dsg1 and anti‐Dsg3 antibodies [5]. Disease severity is assessed using validated tools such as the Pemphigus Disease Area Index [6].

First‐line treatment consists of systemic corticosteroids, often combined with steroid‐sparing immunosuppressants; however, a substantial subset of patients develop refractory disease, particularly with persistent oral lesions. Rituximab, an anti‐CD20 monoclonal antibody that depletes B cells and reduces pathogenic autoantibody production, has emerged as an effective therapy for refractory PV, with randomized controlled trial data supporting its use over conventional immunosuppressants in moderate‐to‐severe disease [7].

We report a case of PV in a 46‐year‐old woman with a 10‐year history of refractory oral lesions that failed to respond to high‐dose corticosteroids, ultimately achieving remission following the initiation of rituximab.

## 2 Case Report

### 2.1 History

**FIGURE 1:**
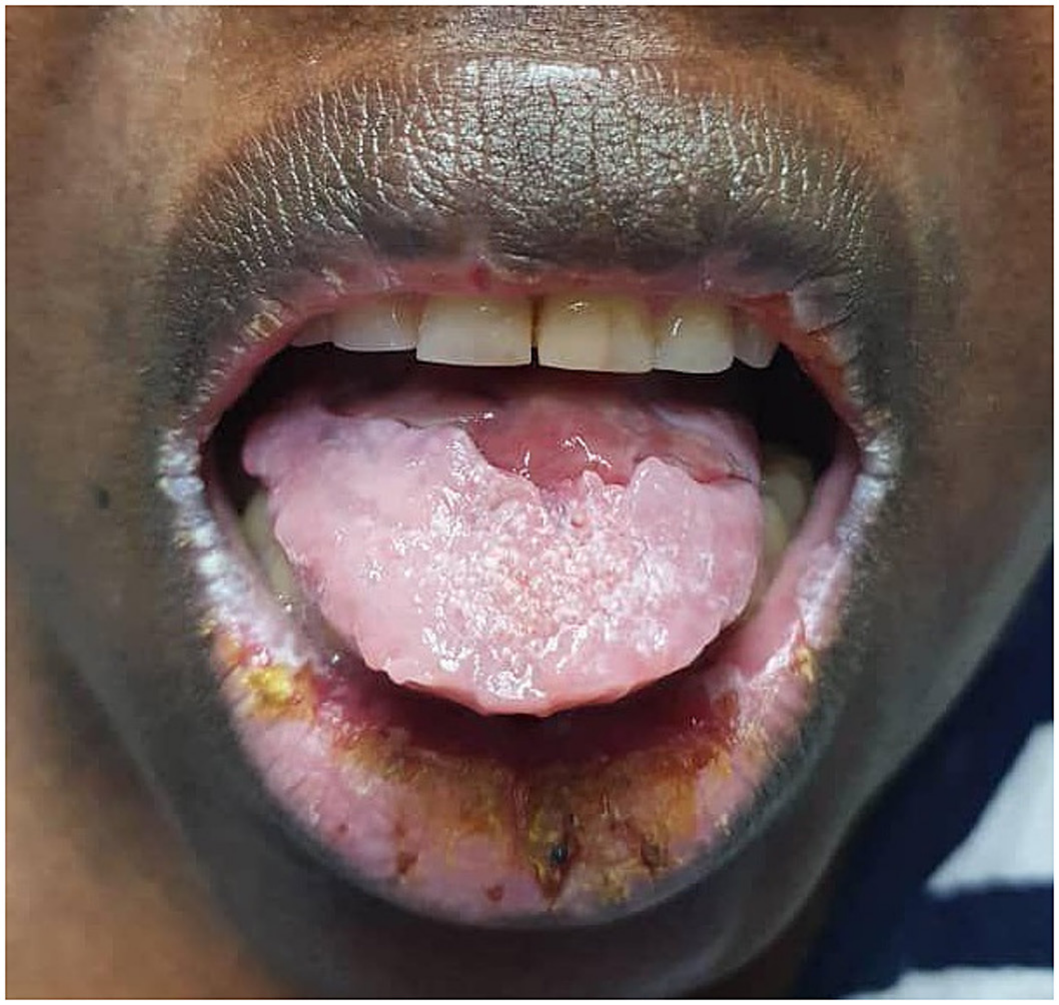
Ulcerative lesion on the dorsum of the tongue.

**FIGURE 2:**
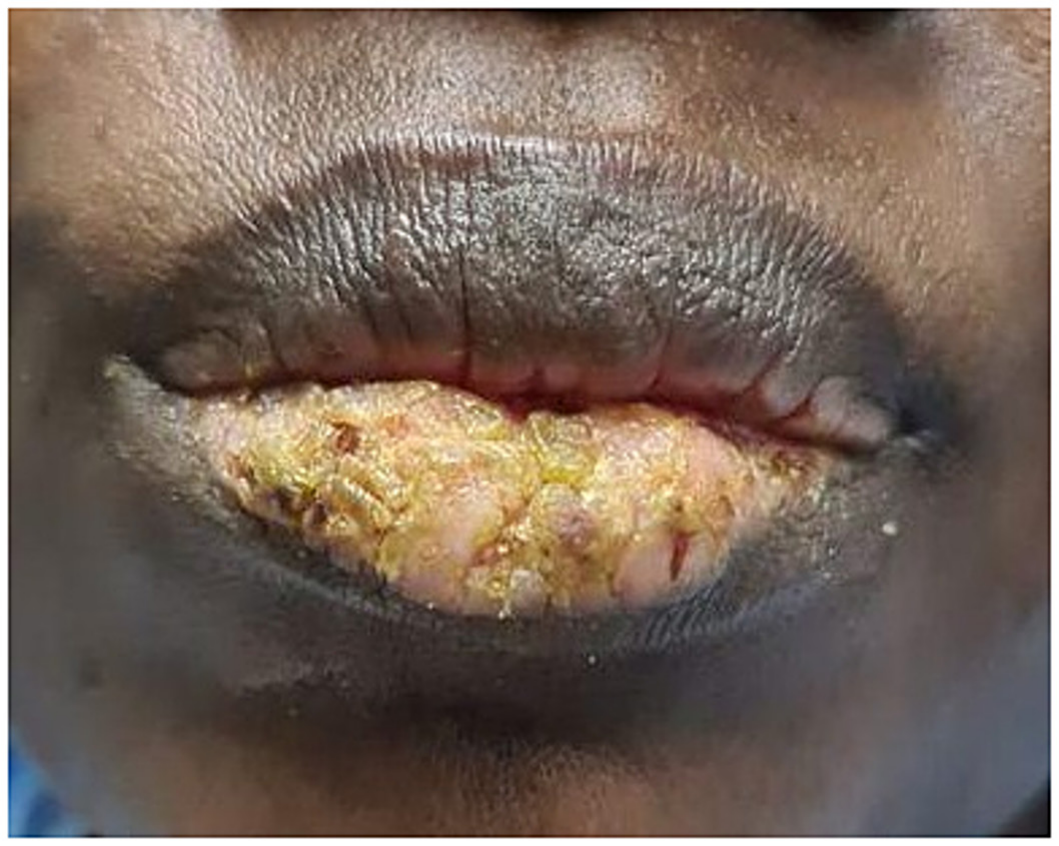
Ulceration with crusting of the lower lip.

A 46‐year‐old female patient presented with a 10‐year history of flaccid blisters, many of which ruptured, forming painful erosions and ulcerations involving the trunk and limbs, associated with severe and persistent oral mucosal disease. Oral involvement predominantly affected the tongue (Figure 1) and lower lip (Figure 2), causing significant pain, impaired oral intake, and resultant functional and nutritional compromise.

At initial presentation, clinical examination revealed extensive desquamated, lichenoid‐appearing patches over the trunk, back, thighs, and face, with scattered intact flaccid blisters and prominent oral mucosal erosions.

### 2.2 Histopathology and Diagnosis

**FIGURE 3:**
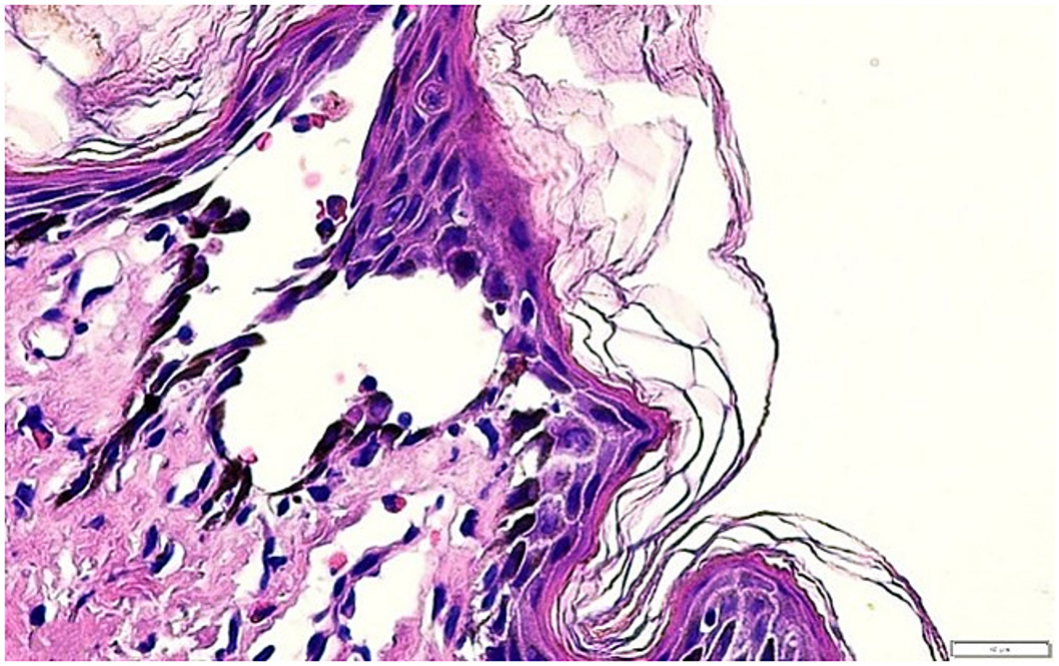
Early vesicle formation characterized by suprabasilar keratinocyte acantholysis and tombstone‐like appearance of residual basal cells (x40 magnification).

**FIGURE 4:**
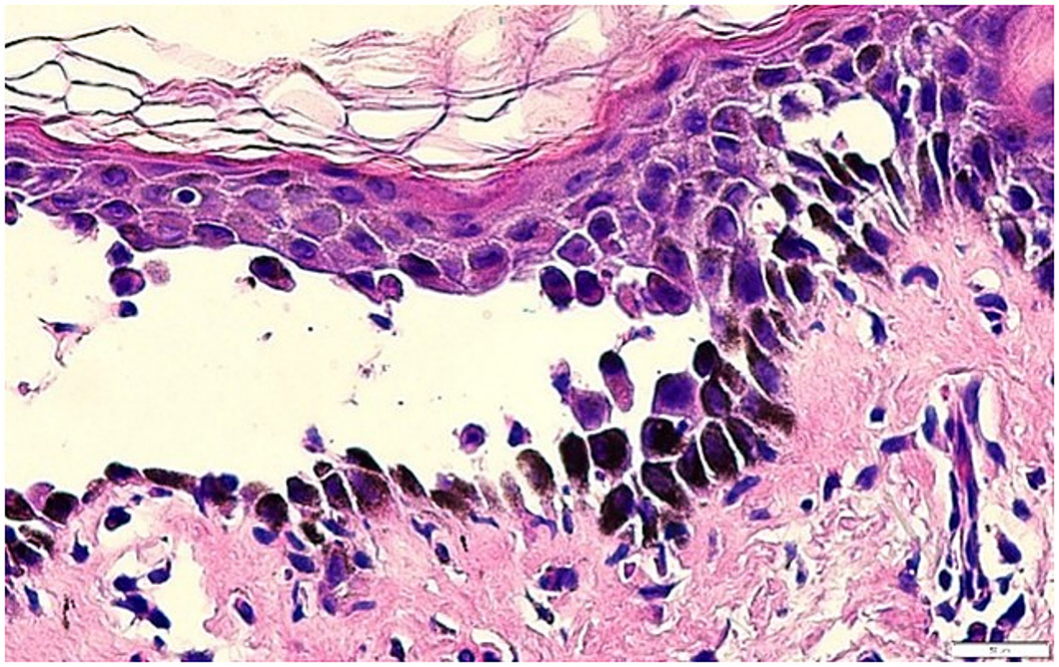
Intraepidermal blister with acantholysis of keratinocytes and suprabasilar tombstone appearance of the basal cells (x40 magnification).

Incisional biopsies obtained from perilesional skin demonstrated suprabasal acantholysis with intraepidermal vesicle formation on hematoxylin and eosin staining (Figures 3 and 4). Direct immunofluorescence findings were consistent with pemphigus vulgaris. An oral mucosal biopsy supported the same diagnosis.

### 2.3 Treatment

The patient was commenced on high‐dose systemic corticosteroids (prednisone 1 mg/kg/day), resulting in complete resolution of cutaneous lesions. However, the oral mucosal disease persisted and remained refractory (Table 1). Repeated attempts to taper systemic corticosteroids resulted in immediate disease flares, leading to long‐term corticosteroid dependence and associated adverse effects.

**TABLE 1:** Summary of treatment administered.

Treatment	Dose	Duration	Response
Prednisone	1 mg/kg/day	10 months	Complete resolution of cutaneous lesions; oral mucosal disease persisted and remained refractory
Azathioprine	2 mg/kg/day—divided doses	6 months	Inadequate clinical response; discontinued
Mycophenolate mofetil	500 mg daily	10 months	Inadequate clinical response; discontinued
Rituximab	1000 mg IV × 2 infusions, 2 weeks apart	Induction phase	Marked clinical improvement of oral mucosal disease; corticosteroids successfully tapered and discontinued
Rituximab	500 mg IV	Every 6 months (ongoing)	Sustained improvement; healing oral erosions; minimal residual erythema; no cutaneous recurrence; nutritional status restored

Multiple steroid‐sparing immunosuppressive agents were trialed over several years. Azathioprine was administered at therapeutic doses on two separate occasions but was discontinued due to inadequate clinical response. Mycophenolate mofetil was subsequently introduced and continued for an extended period, again without satisfactory control of mucosal disease. Despite combination therapy, the patient continued to exhibit persistent oral erosions and ulcerations (Table 1).

Given the recalcitrant disease course, significant morbidity, and failure of conventional immunosuppressive therapy, treatment with rituximab was initiated. Pre‐treatment evaluation, including renal and liver function tests, hepatitis viral screening, human immunodeficiency virus testing, and tuberculosis screening, was unremarkable. Age‐appropriate vaccinations were administered prior to therapy.

Rituximab was administered according to the rheumatoid arthritis protocol, consisting of two intravenous infusions of 1000 mg given two weeks apart as induction therapy.

### 2.4 Results

**FIGURE 5:**
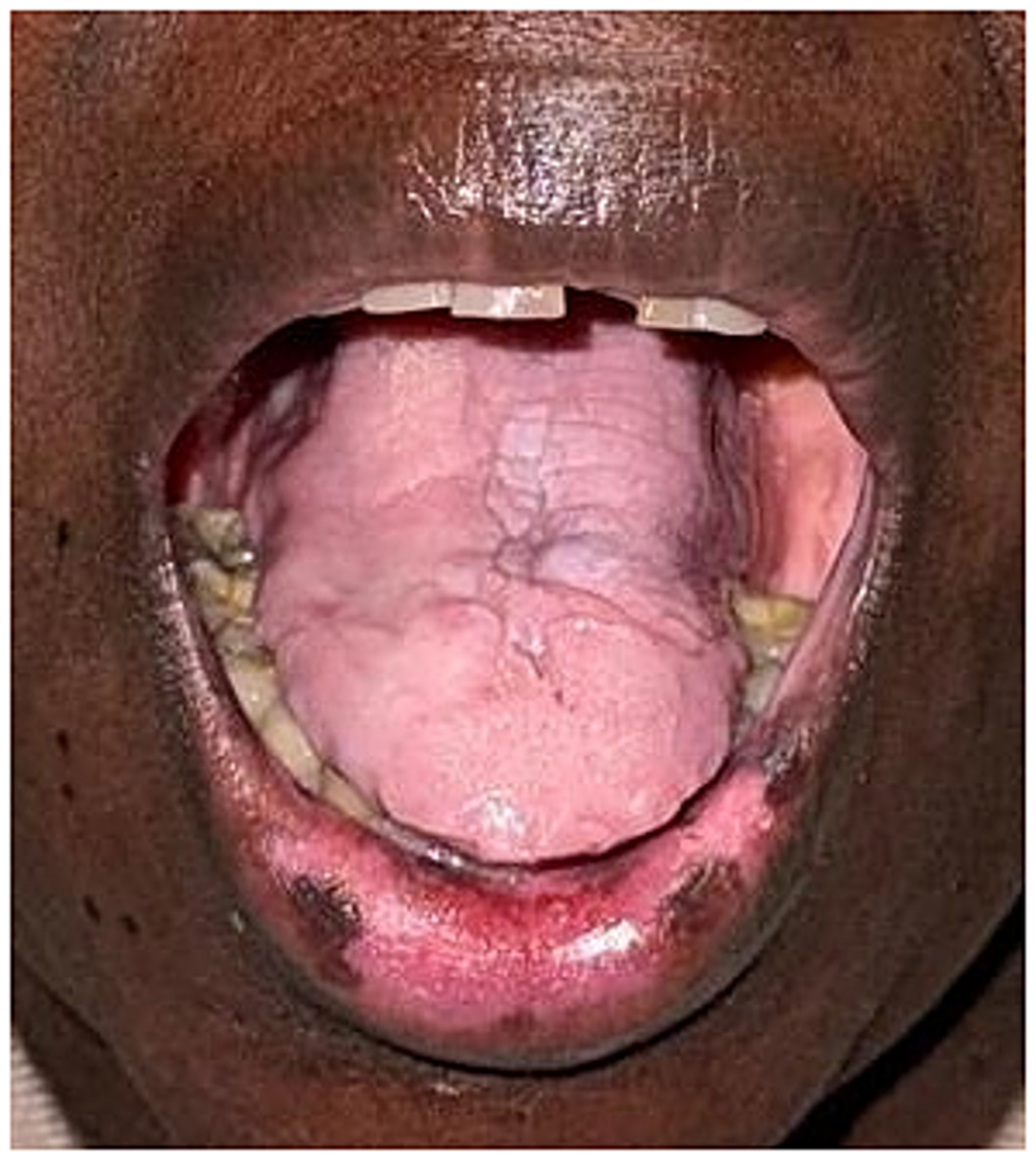
Healed lesion on the dorsum of the tongue.

**FIGURE 6:**
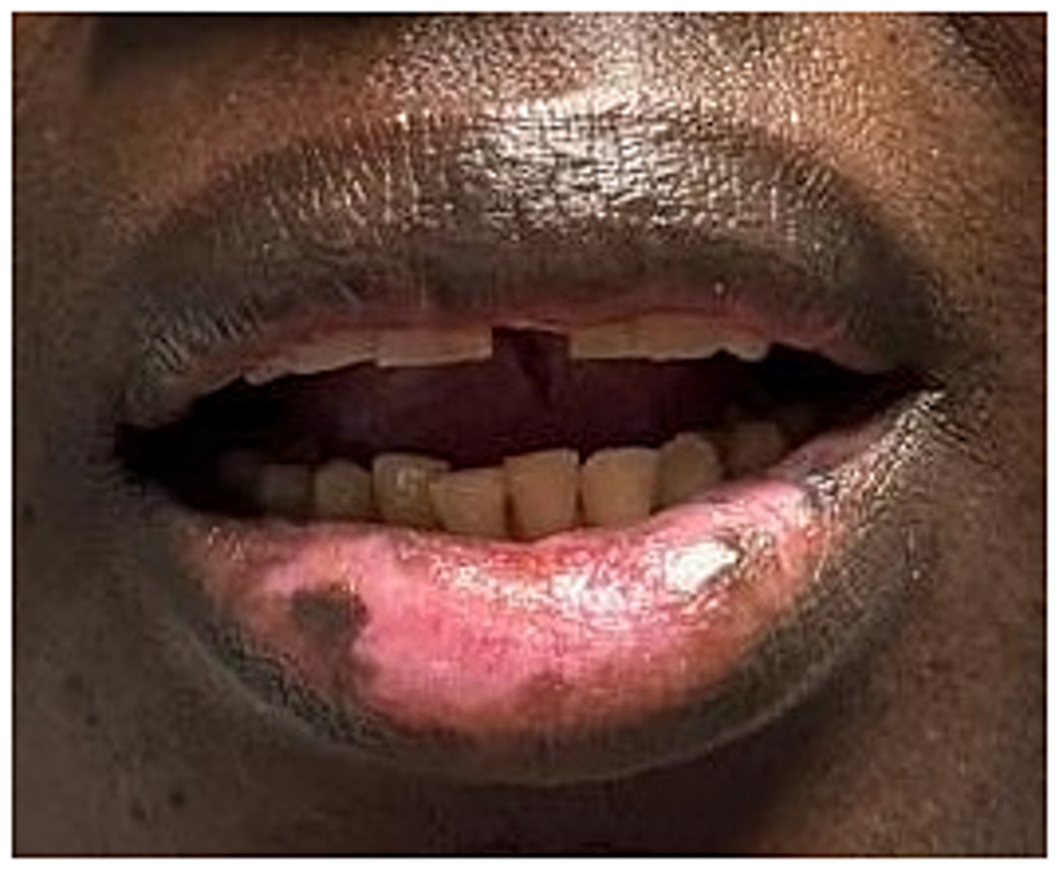
Healed lower lip.

This resulted in marked clinical improvement of the oral mucosal disease (Figures 5 and 6). Systemic corticosteroids were progressively tapered and successfully discontinued by completion of the induction phase.

The patient was subsequently commenced on rituximab maintenance therapy (Table 1), consisting of additional infusions of 500 mg administered at six‐monthly intervals. At follow‐up, she demonstrated sustained clinical improvement, with healing oral erosions, minimal residual erythema, and no recurrence of cutaneous lesions. Her ability to eat improved significantly, with resolution of nutritional compromise.

## 3 Discussion

The therapeutic management of pemphigus is centered on immunosuppression aimed at preventing new lesion formation, promoting healing of existing erosions, and minimizing treatment‐related adverse effects [2]. Corticosteroids remain the cornerstone of first‐line therapy, administered as monotherapy or combined with adjuvant steroid‐sparing agents such as azathioprine or mycophenolate mofetil, with treatment intensity guided by disease severity [1].

Rituximab, a chimeric anti‐CD20 monoclonal antibody, has emerged as the most effective therapy for pemphigus vulgaris, particularly in refractory disease. By inducing rapid B‐cell depletion through antibody‐dependent cellular cytotoxicity, complement‐mediated lysis, and apoptosis, rituximab disrupts the production of pathogenic anti‐desmoglein autoantibodies, thereby reducing acantholysis and mucocutaneous erosions [8]. Its superiority over conventional immunosuppression was definitively established in the Ritux 3 RCT, in which 89% of patients receiving rituximab achieved complete remission off therapy at 24 months, compared with 34% in the prednisone‐only group [7]. Long‐term observational data have corroborated these findings, with remission rates of 70%–90% sustained over several years, attributed to prolonged B‐cell depletion and repopulation with naive, less autoreactive B cells [9, 10]. Based on this evidence, current European Dermatology Forum and International Pemphigus Committee guidelines recommend rituximab as the preferred first‐line therapy for moderate‐to‐severe PV and the treatment of choice in refractory disease [11].

A clinically important additional benefit is rituximab's steroid‐sparing effect, which significantly reduces cumulative corticosteroid exposure and its associated metabolic, musculoskeletal, cardiovascular, and psychological complications—a consideration of particular relevance in corticosteroid‐dependent patients such as the present case. The safety profile is generally favorable; infusion‐related reactions are typically mild and transient, and long‐term data confirm that repeated cycles are well tolerated with low rates of serious adverse events [12]. Pre‐treatment screening for hepatitis B reactivation, hypogammaglobulinemia, and latent infections is nonetheless recommended [12].

## 4 Conclusion

The present case illustrates the therapeutic challenges of mucosal‐dominant PV, in which oral lesions—more resistant to treatment than cutaneous manifestations and a major contributor to morbidity and impaired quality of life—persisted for over a decade despite systemic corticosteroids, azathioprine, and mycophenolate mofetil. Sustained disease control was achieved only following the initiation of rituximab, with rapid and marked improvement in both mucosal and cutaneous disease activity, enabling complete cessation of systemic corticosteroids. This outcome is consistent with published evidence and reinforces rituximab's role as a paradigm‐shifting, targeted therapeutic option that offers durable remission while minimizing corticosteroid dependence in refractory pemphigus vulgaris [1, 2, 11].

## Author Contributions

**Mahlatse Cordelia Kgokolo:** conceptualization, resources, writing – original draft, writing – review and editing. **Lesedi Makgwethele Nevondo:** investigation, validation. **Michaela Beetge:** writing – review and editing. **Razia A. G. Khammissa:** conceptualization, project administration, writing – original draft, writing – review and editing.

## Funding

The authors have nothing to report.

## Consent

Written informed consent was obtained from the patient to publish this report.

## Conflicts of Interest

The authors declare no conflicts of interest.

## Data Availability

The authors have nothing to report.

## References

[1] A. M. Malik , S. Tupchong , S. Huang , A. Are , S. Hsu , and K. Motaparthi , “An Updated Review of Pemphigus Diseases,” Medicina 57, no. 10 (2021): 1080. 34684117 10.3390/medicina57101080 PMC8540565

[2] R. S. Geng and R. G. Sibbald , “Pemphigus Vulgaris: Clinical Aspects and Treatments,” Advances in Skin & Wound Care 38 (2022): 10.1097. 10.1097/ASW.0000000000000307 40184525

[3] A. M. Porro , C. A. Seque , and M. C. C. Ferreira , “Pemphigus vulgaris,” Anais Brasileiros de Dermatologia 94 (2019): 264–278. 31365654 10.1590/abd1806-4841.20199011 PMC6668932

[4] S. S. Venugopal and D. F. Murrell , “Diagnosis and Clinical Features of Pemphigus Vulgaris,” Dermatologic Clinics 29, no. 3 (2011): 373–380. 21605802 10.1016/j.det.2011.03.004

[5] N. van Beek , D. Zillikens , and E. Schmidt , “Diagnosis of Autoimmune Bullous Diseases,” Journal der Deutschen Dermatologischen Gesellschaft = Journal of the German Society of Dermatology: JDDG 16, no. 9 (2018): 1077–1091. 10.1111/ddg.13637 30179336

[6] K. Harman , K. E. Harman , D. Brown , et al., “British Association of Dermatologists' Guidelines for the Management of Pemphigus Vulgaris 2017,” British Journal of Dermatology 177, no. 5 (2017): 1170–1201. 29192996 10.1111/bjd.15930

[7] P. Joly , M. Maho‐Vaillant , C. Prost‐Squarcioni , et al., “First‐Line Rituximab Combined With Short‐Term Prednisone Versus Prednisone Alone for the Treatment of Pemphigus (Ritux 3): A Prospective, Multicentre, Parallel‐Group, Open‐Label Randomised Trial,” Lancet 389, no. 10083 (2017): 2031–2040. 28342637 10.1016/S0140-6736(17)30070-3

[8] J. C. Edwards , L. Szczepanski , J. Szechinski , et al., “Efficacy of B‐Cell–Targeted Therapy With Rituximab in Patients With Rheumatoid Arthritis,” New England Journal of Medicine 350, no. 25 (2004): 2572–2581. 15201414 10.1056/NEJMoa032534

[9] S. Cao , B. Yang , Z. Wang , et al., “Efficacy, Safety, and B‐Cell Depletion Capacity of Three Rituximab Dosing Regimens in the Treatment of Moderate‐To‐Severe Pemphigus Vulgaris and Pemphigus Foliaceus: A 52‐Week Clinical Trial,” Journal of the American Academy of Dermatology 93 (2025): 634–643. 40374120 10.1016/j.jaad.2025.05.1374

[10] V. P. Werth , P. Joly , D. Mimouni , et al., “Rituximab Versus Mycophenolate Mofetil in Patients With Pemphigus Vulgaris,” New England Journal of Medicine 384, no. 24 (2021): 2295–2305. 34097368 10.1056/NEJMoa2028564

[11] D. F. Murrell , S. Peña , P. Joly , et al., “Diagnosis and Management of Pemphigus: Recommendations of an International Panel of Experts,” Journal of the American Academy of Dermatology 82, no. 3 (2020): 575–585. 29438767 10.1016/j.jaad.2018.02.021 PMC7313440

[12] S.‐H. Liu , S.‐H. Wang , and Y.‐G. Zuo , “Efficacy and Safety of Low Dose Rituximab in Pemphigus: An Updated Systematic Review and Meta‐Analysis,” Frontiers in Immunology 16 (2025): 1605243. 40787442 10.3389/fimmu.2025.1605243 PMC12331757

